# Fees Payable to Medical Men as Witnesses

**Published:** 1908-07-25

**Authors:** 


### Medico-Legal Points.

### FEES PAYABLE TO MEDICAL MEN AS WITNESSES.

In one respect, at any rate, the claim of medical practitioners to remuneration for their services stands at present on an unsatisfactory footing. They are often required to give evidence in courts of justice, and complaints are frequent of the inadequate recompense received for loss of time and for out-of-pocket expenses besides. The scale of remuneration varies in different courts, but never errs on the side of liberality, and what may in theory be allowed is frequently reduced by taxing officers who are practically irresponsible, inasmuch as there is no effective means of appealing from their autocratic decisions.

The only cases in which adequate remuneration can be secured are those rare ones in which the witness can make a bargain beforehand as to what his fees are to be, and even then it is sometimes found that supposed bargains cannot be enforced. The hardest cases are those in which evidence has to be given in criminal courts, where the witness may have to attend *de die in diem* for a fee not exceeding one guinea per day, without any allowance for the expense of providing a substitute to attend to his practice during his absence on public duty. Power to fix the scale of fees allowable in criminal courts was given to the Home Secretary in 1851 (14 & 15 Vict. c. 55, s. 5), and under it the scale now in force was issued in February 1858. At present it certainly requires revision. In Scotland a more liberal scale prevails.

In the Chancery Division and King's Bench Division of the High Court professional men, if resident in the town in which the cause is tried are entitled to £1 1s. per diem, and if resident at a distance from the place of trial, £2 2s. to £3 3s. If witnesses attend in one cause only, they are entitled to the full allowance; if they attend in more than one cause, they are entitled to a proportionate part in each cause only. The travelling expenses of witnesses are allowed according to the sums reasonably and actually paid, but in no case shall exceed 1s. per mile one way.

Although the above are the strict rules in the Chancery Division and King's Bench Division of the High Court, yet in practice a somewhat more liberal scale of allowances is usually adopted. Order 65, Rule 27 (9) provides that "such just and reasonable charges and expenses as appear to have been properly incurred in procuring evidence, and the attendance of witnesses, are to be allowed. Allowances to witnesses for hotel expenses on attendance at the trial are in the discretion of the taxing master" (1895, 2 Ch. 514).

In *Wiltshire* v. *Marshall* (1866, W.N. 80), a witness, who was an auctioneer and valuer (and such witness is deemed a professional witness as much as a medical man or barrister), had given evidence on behalf of the plaintiff, and the defendants having given notice of their intention to cross-examine, the witness had been served by the plaintiff with a subpœna to attend again on a certain day. He was in all kept waiting eight days, and upon being called by the defendants refused to be sworn until his expenses were paid. After some discussion in court as to the proper amount, and after having communicated with the taxing master, who referred to the case of *Brocas* v. *Lloyd* (4 W.R. 540), Vice-Chancellor Wood ruled that the proper allowance for the witness would be two guineas a day, being one guinea for his maintenance and another for his loss of time and business. Inasmuch, however, as one of the days during which he had been detained was Sunday, only one guinea for maintenance could be allowed for that day. This made 17 guineas, which together with the witness's first-class railway fare for the double journey from his place of residence amounted together to £19 7s. In *Working Men's Mutual Society* (21 Ch. D. 831) it was held that an auctioneer summoned to give evidence before a special examiner appointed under proceedings in the Chancery Division is, as a professional witness, entitled to be paid a guinea a day by way of compensation for his loss of time, together with the amount of his travelling expenses, if any, including therein his railway fare, first-class from his residence to the place of his examination and back again. And, although sworn, he may refuse to answer any questions until a sum sufficient to cover these amounts has been paid him.

It seems that it is not absolutely necessary that the expenses should be tendered at the time of serving the subpœna, but that if they be tendered to the witness a reasonable time before he is required to appear it is sufficient (*Whiteland* v. *Grant*, 4 Jur 1061). The amount of expenses should be calculated according to the witness's station in life and the circumstances under which he is placed, and the distance of his residence from the place of trial (*Dixon* v. *Lee*, 3 Dowl. 259). If the witness be of such a profession that from the nature of his avocation he cannot find a substitute, the taxing master will allow a reasonable compensation for his loss of time. Thus he will allow payment for loss of time to physicians, surgeons, and solicitors, but not in general to any other professional or scientific men. A professional witness is entitled to his expenses on the scale allowed to persons of his profession, even if he is not called to give professional evidence. Chief Justice Erle, in *Parkinson* v. *Atkinson* (31 L.J., C.P. 199), said: "We do not approve of the rule which is said to prevail in criminal cases, that if a surgeon is called to give evidence not of a professional character, he is only to have the expenses of an ordinary witness. We think the Master was quite right in allowing the expenses of this witness on the higher scale."

Judges differ as to the meaning of "expenses"; some hold expenses to include an allowance according to scale for loss of time, others that "expenses" simply means travelling expenses, and that allowances for loss of time must be claimed in court before being sworn. The latter seems the better course, as a litigant could not reasonably be expected to determine what the allowance should be.

A witness actually appearing cannot be attached for refusing to give evidence before his expenses are paid or tendered (*Newton* v. *Harland*, 1 M. & G. 956). And he may recover from the person on whose behalf he is subpœnaed not only his bare expenses, but such remuneration as is provided for by the scale. No action, however, lies against the solicitor by whom he was subpœnaed, unless the solicitor has expressly contracted to render himself personally liable (*Lee* v. *Everest*, 26 L.J. Ex. 334). In the County Courts the scale of allowances to professional men is from 15s. to £1 1s. per diem. Expert scientific witnesses in the County Court are allowed £1 1s. to £3 3s. for qualifying to give evidence if costs are taxed on B scale, and from £1 1s. to £5 5s. if taxed on C scale. Attending court on trial per diem, £1 1s. to £2 2s. on B scale, and £1 1s. to £3 3s. on C scale.

By the regulations made by the Secretary of State with regard to witnesses in criminal prosecutions, dated June 14, 1904, there may be allowed to practising members of the legal and medical professions, for attending to give professional evidence, but not otherwise, allowance not exceeding the sums stated in the following scale: For attending to give evidence in the town or place where the witness resides or practises; if the witness attends to give evidence in one case only, not more than one guinea per diem; if the witness gives evidence on the same day in two or more separate and distinct cases, not more than two guineas. For attending to give evidence elsewhere than in any town or place where the witness resides or practises, whether in one or more cases, not more than two guineas per diem. In this regulation "town" means municipal borough or urban district, and "place" means within a radius of three miles from the court at which the witness attends to give evidence. For attending court from a distance of over two miles there may be allowed:—

(1) To witnesses travelling by railway or other public conveyance, the fare actually paid. Railway fares, except for special reasons, allowed by the court shall be third class; and if return tickets are available, only return rates shall be allowed. (2) Where no railway or other public conveyance is available and one or more witnesses necessarily travel by a hired vehicle, the sum actually paid for the hire of such vehicle, not exceeding 1s. a mile each way: provided that, where two or more witnesses attend from the same place, the total allowance shall not exceed 1s. a mile each way, unless the court is satisfied that it was reasonably necessary to hire more than one vehicle. (3) To each witness travelling on foot or by a private conveyance, where no railway or other public conveyance is available, a sum not to exceed 2d. a mile each way.

On an examination before a magistrate there may be allowed to prosecutors or witnesses, being members of the medical profession, if resident in the city, borough, or place where the examination is taken, or within a distance not exceeding two miles from such place, for their loss of time and trouble in attending to give professional evidence on such examination, but not otherwise, a sum, in the discretion of the magistrate, for each attendance, not to exceed 10s. 6d. If such prosecutor or witness reside elsewhere, then a sum, for the same, not to exceed one guinea. For mileage a sum not to exceed 3d. per mile each way.

In criminal cases no tender of fees is necessary, except in the case of witnesses living in one distinct part of the United Kingdom being required to attend subpœnaes directing their attendance in another, who are not liable to punishment for disobedience of the process, unless at the time of service a reasonable and sufficient sum of money to defray their expenses in coming, attending, and returning have been tendered to them.

